# Supplementary material for: An integrative systematic review on interventions to improve layperson’s ability to identify trustworthy digital health information
Source: PLOS Digit Health. 2024 Oct 25;3(10):e0000638. doi: 10.1371/journal.pdig.0000638 (PMC11508166; doi:10.1371/journal.pdig.0000638)
Supplement: S1 Checklist — (DOCX) [file pdig.0000638.s001.docx]

**S1 Checklist:** **Quality assessment (Mixed method appraisal tool checklist) version 2018** [46]

| **Citation** | **SCREENING QUESTIONS** | | **RANDOMIZED CONTROLLED TRIALS** | | | | | | **Total score** |
| --- | --- | --- | --- | --- | --- | --- | --- | --- | --- |
|  | **Are there clear research questions?** | **Do the collected data allow to address the research questions?** | **Is randomization appropriately performed?** | **Are the groups comparable at baseline?** | **Are there complete outcome data?** | | **Are outcome assessors blinded to the intervention provided?** | **Did the participants adhere to the assigned intervention?** |  |
| [37] | Yes | Yes | No | Yes | Yes | | No | Yes | **60% quality criteria met** |
| [2] | Yes | Yes | Yes | Yes | Yes | | No | No | **60% quality criteria met** |
| [10] | Yes | Yes | Yes | Yes | Yes | | No | Yes | **80% quality criteria met** |
| [39] | Yes | Yes | Yes | Yes | Yes | | No | Yes | **80% quality criteria met** |
| [40] | Yes | Yes | Yes | Yes | Yes | | No | Yes | **80% quality criteria met** |
| [44] | Yes | Yes | No | Yes | Yes | | No | Yes | **60% quality criteria met** |
| [43] | Yes | Yes | No | Yes | Yes | | No | Yes | **60% quality criteria met** |
|  |  |  | **QUANTITATIVE DESCRIPTIVE STUDIES** | | | | | |  |
|  |  |  | **Is the sampling strategy relevant to address the research question?** | **Is the sample representative of the target population?** | **Are the measurements appropriate?** | | **Is the risk of nonresponse bias low?** | **Is the statistical analysis appropriate to answer the research question?** |  |
| [9] | Yes | Yes | Yes | No | Yes | | Yes | Yes | **80% quality criteria met** |
| [38] | Yes | Yes | Yes | Yes | Yes | | Yes | Yes | **100% quality criteria met** |
| [41] | Yes | Yes | No | Yes | Yes | | Yes | Yes | **80% quality criteria met** |
| [45] | Yes | Yes | Yes | Yes | Yes | | Yes | No | **80% quality criteria met** |
|  |  |  | **NON-RANDOMIZED STUDIES** | | | | | |  |
|  |  |  | **Are the participants representative of the target population?** | **Are measurements appropriate regarding both the outcome and intervention (or exposure)?** | **Are there complete outcome data?** | **Are the confounders accounted for in the design and analysis?** | | **During the study period, is the intervention administered (or exposure occurred) as intended?** |  |
| [42] | Yes | Yes | Yes | Yes | Yes | | Yes | Yes | **100% quality criteria met** |
