# Supplementary material for: An integrative systematic review on interventions to improve layperson’s ability to identify trustworthy digital health information
Source: PLOS Digit Health. 2024 Oct 25;3(10):e0000638. doi: 10.1371/journal.pdig.0000638 (PMC11508166; doi:10.1371/journal.pdig.0000638)
Supplement: S1 Table — (DOCX) [file pdig.0000638.s003.docx]

**S1 Table:** **Search strategy**

| **Database** | **Search terms** | **Result** |
| --- | --- | --- |
| **Ovid Medline** | (adult OR adults) OR (patient OR patients) OR (layperson OR laypersons) OR (caregiver OR caregivers) | 8008770 |
|  | (Intervention OR interventions) OR Educational programs OR (health literacy And curriculum) OR Community outreach OR Interactive workshops OR (Online portal OR Patient Portals) | 1230788 |
|  | Information seeking behaviour OR consumer health information OR online information OR social media OR Access to information | 28822 |
|  | (adult OR adults) OR (patient OR patients) OR (layperson OR laypersons) OR (caregiver OR caregivers) AND (Intervention OR interventions) OR Educational programs OR (health literacy And curriculum) OR Community outreach OR Interactive workshops OR (Online portal OR Patient Portals) AND Information seeking behaviour OR consumer health information OR online information OR social media OR Access to information | **1545** |
| **Cochrane library** | "adult"OR "patient" OR "layperson"OR "carer" | 1393390 |
|  | ("intervention"OR "Interventions") OR "educational programs" OR "Health literacy curriculum" OR "community outreach" OR "interactive workshops" OR "Online portal" | 522206 |
|  | "access to information" OR (“information seeking" OR "information seeking behavior") OR ("consumer health information" OR "patient health information" OR "public health information") OR ("internet information” OR "online information") OR ("Social media" OR "online system" ) | 4321 |
|  | ("adult") OR ("patient") OR ("layperson") OR ("carer") AND ("intervention”OR "Interventions") OR "educational programs" OR "Health literacy curriculum" OR "community outreach" OR "interactive workshops" OR "Online portal" AND "access to information" OR (“information seeking" OR "information seeking behavior") OR ("consumer health information" OR "patient health information" OR "public health information") OR ("internet information” OR "online information") OR ("Social media" OR "online system") | **2073** |
| **Embase database** | ('adult' OR 'patient' OR 'public' OR 'caregiver') | 14,886,182 |
|  | ('information seeking' AND 'behavior' OR 'consumer health information' OR ('access' AND 'medical information') OR ('medical information' AND ('internet' OR 'online system') | 33, 484 |
|  | ('intervention' OR 'interventions' OR 'learning strategy' OR 'community outreach' OR ('health literacy' AND 'curriculum') OR 'collaborative learning' OR 'web-based intervention') | 1,814,297 |
|  | ('adult' OR 'patient' OR 'public' OR 'caregiver') AND ('information seeking' AND 'behavior' OR 'consumer health information' OR ('access' AND 'medical information') OR ('medical information' AND ('internet' OR 'online system'))) AND ('intervention' OR 'interventions' OR 'learning strategy' OR 'community outreach' OR ('health literacy' AND 'curriculum') OR 'collaborative learning' OR 'web-based intervention') | **3,229** |
| **Academic Search Complete** | (adults or adult or middle aged) OR sick people OR ( layperson or public ) OR ( carers or caregivers ) | 3,520,733 |
|  | (interventions or strategies) OR educational programs OR health literacy curriculum OR interactive learning OR online platform OR community outreach programs | 2,234,453 |
|  | health information seeking behavior OR patient health information OR online information seeking OR internet use OR access to information | 81,368 |
|  | (adults or adult or middle aged) OR sick people OR (layperson or public) OR ( carers or caregivers ) AND (interventions or strategies ) OR educational programs OR health literacy curriculum OR interactive learning OR online platform OR community outreach programs AND health information seeking behavior OR patient health information OR online information seeking OR internet use OR access to information | **3,646** |
| **APApschinfo** | ( adults or adult ) OR patient OR laypeople OR ( carers or caregivers or family members or relatives or informal carers ) | 74,177) |
|  | (interventions or strategies or best practices) OR ( educational programs or interventions ) OR health literacy curriculum OR ( community outreach or community program or community engagement ) OR interactive workshops OR patient portal | 883,116 |
|  | (information-seeking behavior or information needs or information seeking strategies) OR consumer health information OR online information search OR social media use OR access to information | 1,095 |
|  | ( adults or adult ) OR patient OR laypeople OR ( carers or caregivers or family members or relatives or informal carers ) AND ( interventions or strategies or best practices ) OR ( educational programs or interventions ) OR health literacy curriculum OR ( community outreach or community program or community engagement ) OR interactive workshops OR patient portal AND (information-seeking behaviour or information needs or information seeking strategies ) OR consumer health information OR online information search OR social media use OR access to information | **3,772** |
