# Supplementary material for: An integrative systematic review on interventions to improve layperson’s ability to identify trustworthy digital health information
Source: PLOS Digit Health. 2024 Oct 25;3(10):e0000638. doi: 10.1371/journal.pdig.0000638 (PMC11508166; doi:10.1371/journal.pdig.0000638)
Supplement: S2 Table — (DOCX) [file pdig.0000638.s004.docx]

**S2 Table: Number of participants of included studies**

| **Number of participants** | **Number of studies n (%)** |
| --- | --- |
| < 100 | 7 (58.4) [2,9,37-41] |
| 100-1000 | 3 (25) [10,42,43] |
| >1000 | 1 (8.3) [4] |
| No information | 1 (8.3) [45] |
