# Supplementary material for: An integrative systematic review on interventions to improve layperson’s ability to identify trustworthy digital health information
Source: PLOS Digit Health. 2024 Oct 25;3(10):e0000638. doi: 10.1371/journal.pdig.0000638 (PMC11508166; doi:10.1371/journal.pdig.0000638)
Supplement: S3 Table — (DOCX) [file pdig.0000638.s005.docx]

**S3 Table:** **Target age group of included studies**

| **Age group** | **Number of studies (%)** |
| --- | --- |
| Young adults | 8 (66.6) [2,10,37-39,42-44] |
| Young and old adults | 1 (8.3) [41] |
| Old adults | 3 (25) [9,40,45] |
