# Supplementary material for: An integrative systematic review on interventions to improve layperson’s ability to identify trustworthy digital health information
Source: PLOS Digit Health. 2024 Oct 25;3(10):e0000638. doi: 10.1371/journal.pdig.0000638 (PMC11508166; doi:10.1371/journal.pdig.0000638)
Supplement: S4 Table — (DOCX) [file pdig.0000638.s006.docx]

**S4 Table****: Study design of included studies**

| **Study design** | **Number of studies (%)** |
| --- | --- |
| quasi-experimental | 1 (8.3) [42] |
| Quantitative | 4 (33.3) [9,38,41,45] |
| Randomised control trial | 7 (58.4) [2,10,37,39,40,43,44] |
