# Supplementary material for: An integrative systematic review on interventions to improve layperson’s ability to identify trustworthy digital health information
Source: PLOS Digit Health. 2024 Oct 25;3(10):e0000638. doi: 10.1371/journal.pdig.0000638 (PMC11508166; doi:10.1371/journal.pdig.0000638)
Supplement: S5 Table — (DOCX) [file pdig.0000638.s007.docx]

**S5 Table****: Countries of included studies**

| **Country** | **Number of studies (%)** |
| --- | --- |
| Germany | 1 (8.3) [37] |
| Australia | 1 (8.3) [38] |
| USA | 6 (50) [40-45] |
| Norway | 1 (8.3) [2] |
| Georgia | 1 (8.3) [10] |
| Canada | 1 (8.3) [9] |
| UK | 1 (8.3) [39] |
