# Supplementary material for: An integrative systematic review on interventions to improve layperson’s ability to identify trustworthy digital health information
Source: PLOS Digit Health. 2024 Oct 25;3(10):e0000638. doi: 10.1371/journal.pdig.0000638 (PMC11508166; doi:10.1371/journal.pdig.0000638)
Supplement: S6 Table — (DOCX) [file pdig.0000638.s008.docx]

**S6 Table:** **Years of publication of included studies**

| **Year of publication** | **Number of studies (%)** |
| --- | --- |
| 2006- 2011 | 2 [9,10] |
| 2012-2017 | 4 [2,37,38,45] |
| 2017-2023 | 6 [39-44] |
