# Supplementary material for: An integrative systematic review on interventions to improve layperson’s ability to identify trustworthy digital health information
Source: PLOS Digit Health. 2024 Oct 25;3(10):e0000638. doi: 10.1371/journal.pdig.0000638 (PMC11508166; doi:10.1371/journal.pdig.0000638)
Supplement: S7 Table — (DOCX) [file pdig.0000638.s009.docx]

**S7 Table: Summary of Intervention’s description**

| **Study citation** | **Intervention type** | **Intervention setting and delivery (Context)** | **Intervention style/content/material** | **Comparison (yes/no)** | **Intervention duration** |
| --- | --- | --- | --- | --- | --- |
| [37] | Short source evaluation training intervention | Web-based intervention/All delivered online. | **Style**: web-based, self-paced training (three parts).  **Content:**  1. The first part aimed at raising awareness of the importance of source evaluation during web search.  2. In the second part, individuals learned how to evaluate information sources based on the search results and the actual web pages.  3. The third part of the intervention  addressed the comparison of information across different information sources to identify corroborated, unique, or discrepant information.  **Material**: two web pages were presented that provided discrepant information about Mother Theresa’s day of death | Present/ exposure to Web  Search without having undergone the intervention. | Completing the source evaluation intervention took approximately 20min |
| [45] | Pharmacy Community Outreach  Program | Community-based intervention/ Delivered by  faculty members and the campus librarian. | **Style**: An educational learning series (Three series)  **Content:**  -Participants learn how to search for health information on the Internet, how to find reliable information on health, drugs, and supplements and how evaluate the accuracy and reliability of health information Web sites.  -Participants were instructed on how to use MedlinePlus, a Web site from the National Library of Medicine and the National Institutes of Health, to find health information  -**Material:** Derived from National Institute on Aging’s “Helping Older Adults Search for Health Information Online: A Toolkit for Trainers. | Absent comparison | No information |
| [38] | Community educational initiatives | Library-based intervention/Delivered by a team of clinicians and consumer representatives from a large metropolitan hospital. | **Style:** Integrative workshops (group sessions)  **Content:** No information  **Material:** No information. | Absent comparison | 2 hours group sessions |
| [2] | Web portal | Web-based intervention/All information delivered online, and Participants were sent tasks by email. | **Style:** Interactive online skill-based learning (Three tasks)  **Content:**  First task (searching task): Participants were asked to formulate a question and to answer it by searching for online information from any source including the web portal.  Second task (critical appraisal task): All participants were asked to rate the trustworthiness of an online article using the full DISCERN critical appraisal tool which was provided on the web portal.  Third task: reporting of beliefs about search for health information and activation  final task (satisfaction with the web portal)  **Material:** online article describing how to prevent swine flu, and that included information about vaccinations and alternative therapies. The specific material chosen for evaluation was taken from a health information site identified using a Google search and was typical of the kind of information available on sites used by lay-people searching for health information | Present /No access to the web portal | 3 days to access to web portal followed by  3 weeks |
| [10] | Behavioural intervention | Web-based intervention/ Guided by one male and one female community-based group facilitator, at least one of whom was an HIV-positive peer counsellor. | **Style:** Internet skills-building intervention (eight group sessions)  **Content:**  - The first two sessions included exercises to motivate interest in using the Internet and group activities for searching for information of personal interest. Participants also established e-mail accounts, explored chat rooms and instant messaging, and discussed the opportunities and hazards of online relationships.  - In Session 3, participants were instructed in criteria for evaluating the quality of information obtained online (the PILOT criteria).  - Sessions 4 –7: Participants were instructed in searching for health information, care and support resources, and clinical trials. participants conducted searches of health information, evaluated search results, and role-played taking information to their doctor.  - The final session included reviewing skills associated with developing online social support and strategies for gaining access to the Internet at home and elsewhere.  **Material:** Two HIV webpages (HIV treatment information). One Web page describe the widely accepted HIV/AIDS treatment guidelines: the Journal of the American Medical Association  (JAMA) patient page on drug treatment options for HIV, posted on the www.medem.com Web site. The second Web page made scientifically unsupported claims of a successful treatment or cure for AIDS from goat’s blood serum (http://www.quantumbalancing.com/AIDS_CURE  .htm | Present/Health information support group. | 120-min group sessions that met twice weekly for 4 consecutive weeks. |
| [9] | Public library workshop | Library-based/Delivered by Consumer  health librarians together with the two university-based investigators. | **Style:** internet skill building – intervention  **Content:**  -Introductory Exercise  -Preliminary Unassisted Search  -Introduction to Three Searching Tools  -Search Engines, Online Directories/Indexes, Subject Starters/Gateways)  -Focus on Search Engines  (Searching Tips, Disadvantages of Search Engines, Review of Search Engines, Practice Using Search Engines)  -Focus on Online Directories (Purpose of Directories, Representative General/Library Directories, Representative Medical Directories), Practice Using Directories)  -Focus on Subject Starters (Purpose of Subject Starters, Searching Tips, Examples, Practice Using Subject Starters).  -How to Locate High-Quality Health and Cancer Web sites (Beware of Quackery on the Web).  -Review of Representative  -High-Quality Cancer Web sites  -Wrap-Up Exercise  **Material:** Participants were introduced to health and cancer Web sites of various top-level domains (.gov, .com, .org, .edu) and Web pages accredited by the Health on the Net Foundation , such as the National Cancer Institute and the American Cancer Society | absent comparison | four Internet workshops held on four consecutive months (one workshop/month |
| [39] | Enriching Wikipedia contents | Web-based intervention/Delivered online | **Style:** enriching Wikipedia content with summary tables from level 1 evidence on the effects of care.  **Content:** Posting the relevant Cochrane review’s Summary of Findings table (modified to increase readability) on the target Wikipedia page along with references to the review’s web page and full text.  **Material:** Adding an evidence-table to four Wikipedia pages (trifluoperazine—a less used antipsychotic; chlorpromazine—a old widely used antipsychotic drug; palperidone—an expensive new antipsychotic drug and one important talking therapy—cognitive behavioural therapy). | Present/ Leaving the existing page unmodified | 12 months |
| [40] | e health tutorial | web-based intervention/ guided by a trained facilitator | **Style:** Multimedia learning (group session)  **Content:**  -session 1: Basic computer/Internet terms and skills  -session 2: Introduction to the MedlinePlus.gov website  -session 3: use of the health Topics section on MedlinePlus.gov., Use of the Drugs and Supplements and the Medical Encyclopaedia sections on MedlinePlus.gov  -session 4: How to evaluate the quality of health information websites  **Material:** Tutorial was developed by the National Institute on Aging (NIA) | Present/ a paper-based tutorial | a total of four sessions that occurred twice a week, 2 hours per session, over 2 weeks |
| [44] | Boosting consensus reasoning | Paper-based intervention? /Guided by infographic. | **Style:** Competence-based intervention  **Content:**  Participants were exposed to 10 statements, of which five were scientifically accurate and five were at odds with the best available evidence. Participants responded by indicating the accuracy of a statement. An infographic presenting three steps that can be used to evaluate a claim, was included at the end of T0, T1, and T2. T0: baseline; T1: first follow-up wave; T2: second follow-up wave; T3: final wave. In each subsequent wave, four new statements were added to the list of statements: two accurate ones and two inaccurate ones.  **Material:** Statements were sourced from preprints of early research on public perceptions of COVID-19, public health agencies and medical institutes, media tracking organizations, and expert reports in established media. | present/No infographic provided | 3 weeks |
| [43] | Accuracy nudging intervention | Social media-based intervention/ Delivered online | **Style:** Competence-based intervention  **Content:**  **-**Participants rated the accuracy of a single headline (unrelated to COVID-19) before beginning the news-sharing task. Each participant saw one of four possible headlines.  **Material:** no information | present/ news-sharing task | 3 days? |
| [42] | Educational video | Media-based intervention/ Conducted by an HIV expert nurse clinician | **Style:** Educational video  **Content:** Watching an educational video about identifying a reliable Internet site.  **Material:** The video was developed by national library of medicine | present/ watching video +individual time with an HIV nurse expert | 16-minute (video time) |
| [41] | prior topic knowledge pre-activation support tool | web-based intervention/ Delivered through laptop, running windows 7 and using Google search engine and Google Chrome web browser | **Style:** Problem-based search learning  **Content:**  -Participants performed the semantic pre-activation task prior to the search phase.  -Participants started by reading the search problem statements, then they were instructed to produce three keywords related to a concept extracted from the search problem and to the search problem statement.  -Participants were instructed to imagine three different keywords that might be useful to search for the answer, later on, on the Internet. -This step was repeated three times (i.e. for three different concepts extracted from the search problem statements).  - Participants completed the semantic pre-activation task online before each search problem. Once they had completed the pre-activating task, the experimenter switched to the searching phase on the Internet. The experimenter switched between the pre-activation phase and the search for each search problem one by one  **Material**: Health problems related to how human body functioning | Present/ No intervention | Unlimited time (Participants stop the search at any time when they believed they had found a satisfactory answer) |
