# Supplementary material for: An integrative systematic review on interventions to improve layperson’s ability to identify trustworthy digital health information
Source: PLOS Digit Health. 2024 Oct 25;3(10):e0000638. doi: 10.1371/journal.pdig.0000638 (PMC11508166; doi:10.1371/journal.pdig.0000638)
Supplement: S8 Table — (DOCX) [file pdig.0000638.s010.docx]

**S8 Table: Interventions measures, outcomes, effectiveness, Critique**

| **Intervention type** | **Outcomes / intervention effectiveness** | **Measures of evaluation** |
| --- | --- | --- |
| Short source evaluation intervention  [37] | **-Individuals’ navigation behaviour:** With regard to the time spent on objective web pages, subjective web pages, and commercial web pages, a MANOVA showed a significant main effect of intervention.  **-Post-search decisions:** with regard to the certainty of the decision, participants in the intervention group were significantly more certain of their decision than controls  **-Epistemic beliefs:** after the intervention participants possessed significantly stronger beliefs that Internet-based knowledge claims need to be critically evaluated through cross-checking. | **- MANOVA**  **- X square test**  **-** ISEQ (Internet-Specific Epistemological Questionnaire), a previously tested, reliable questionnaire that includes four items to assess Internet-specific beliefs was used. |
| Pharmacy Community Outreach  Program  [45] | **Primary outcome.**  **Skills:**  -Participants indicated they were very likely to use MedlinePlus to find health information (83%),  -Sixty-five percent strongly agreed they felt more confident in their ability to evaluate the reliability of health information found on the Internet.  **-Knowledge:** improve older adults’ understanding about their health and medications**:** Participants are more likely to use Medline plus to find information on drugs and supplements (79%)  **- Behaviour:** Medication adherence: improve medication adherence  **Secondary outcome.**  **-health outcomes: Hb a 1c and blood pressure: reduction of glycated haemoglobin A1c and blood pressure** | **-Survey tools** |
| Community educational initiatives  [38] | **Primary outcomes:**  **-Knowledge:** most participants strongly agreed (59.6%) or agreed (37.1%), that this workshop improved their knowledge about evidence-based health information.  **-Skills:** most participants strongly agreed (56.8%) or agreed (42.1%) that this workshop improved their skills with finding and using evidence-based health information’.  **-Attitude:** most participants strongly agreed or agreed (78.2%) that the workshop improved their attitude towards evidence-based health information.  **-Behaviour:** 52.8% strongly agreed; 40.5% agreed that attending the workshop would change the way they looked for and used health information in future. | -A two-part paper-based questionnaire was administered to workshop participants pre and post workshops.  -The questionnaire used 5-point Likert scales (Strongly agree, Agree, Neutral, Disagree, strongly disagree) to gather data about the workshop’s impacts on knowledge,  skill, attitude, and behaviour.  - A chi-square test, Mann–Whitney U-test, Kruskal–Wallis test |
| Web portal  [2] | **Primary outcomes:**  -Critical skills (searching task and critical appraisal task): Only minor improvements in the intervention group were identified by both tasks and these differences were not statistically significant.  **-Beliefs about searches for health information and activation**: A statistically significant difference was found for overall attitude towards search in favour of the intervention group.  **-Satisfaction with the web portal**: The satisfaction with the web portal was good. | -The number of research-based sources reported by participants in the searching task  -DISCERN appraisal tool was used for critical appraisal task  -Beliefs about participation were evaluated using a Theory of planned behaviour (TPB) questionnaire, that addressed intention to search and underlying factors, and the PAM questionnaire describing overall activation  -Honeycomb model, a useful instrument applied to measurements of Internet site user experiences was used to measure satisfaction with the portal. |
| Behavioural intervention  [10] | **Primary outcomes**  -Behaviour; Self-efficacy for information seeking significant interaction between assessment time and intervention condition on self-efficacy for health information use. The Internet skills intervention demonstrated greater self-efficacy at each follow-up.  -Skills; Health information evaluation skills  -Internet use/access  - Internet use: there was a significant interaction between intervention condition and assessment time for using the Internet for health, clinical trials, and e-mail, and the interaction effect on bringing information to health care providers was also significant  **Secondary health outcomes:**  -Social support  Planned comparisons showed that the Internet skills-building group reported significantly greater social support at all three follow-ups.  -Affective depression  Participants in the Internet skills intervention reported fewer symptoms of affective depression over time than did the comparison group | -Participants completed four items assessing beliefs to perform specific information-related behaviours. They responded to items on scales ranging from 0 (cannot do at all) to 10 (certain I can do), with scores representing mean responses ranging from 0 to 10.  -Participants rated the Web pages on five dimensions of Web site: quality: accuracy, amount of detail, trustworthiness– credibility, relevance, and usefulness, using 10-point scales ranging from 1 (not at all) to 10 (very).  -Participants indicated the number of times they had used the Internet.  -The Social Support Questionnaire consists of 15 items that assess perceived social support Participants responded to these items on 4-point scales ranging from 1 (definitely true) to 4 (definitely false);  -The Centers for Epidemiological Studies– Depression Scale (CES-D) is a 20-item scale that assesses symptoms of depression over the previous 7 days, with scores ranging from 0 (no days) to 3 (5–7 days |
| Public library workshop  [9] | **Primary outcomes**  -**Usefulness of the workshop information**  Most participants (63.4%) rated the workshop as very useful. 65.1% found the number of topics presented at the workshops to be just right.  Internet Use Questionnaire  **- Ease of Internet searching**  (82.9%) felt comfortable searching independently for Web-based cancer information after the workshop as compared to 36.6% people prior to the workshop.  -**Self-rated understanding of the Internet:** it is much higher after attending the workshop. Thirty-eight participants (92.7%) reported that the workshop helped them to differentiate between search engines and health directories/databases.  - Seventy percent of participants indicated that they would rely on the Internet for cancer information in the future | -Participants were asked to complete questionnaires covering an evaluation of the workshop, and an Internet use that includes Mixed Likert type forced response and open-ended questions, each cover 3 items.  **-** On a scale from 1 (very easy) to 10 (very difficult) used to measure ease of internet searching  - Self-rated understanding of the Internet on a scale from (1 = poor understanding; 5 = excellent understanding) |
| Enriching Wikipedia contents [39] | **Coprimary outcomes**  1. The number of visits to the free summary page (all page views).  2. The number of full-text downloads  -The point estimates for the ratio of geometric means favoured the intervention group for coprimary outcomes.  **Secondary outcomes**  -Altimetric score indicated some evidence of an intervention effect (statistically significant. -Enriching Wikipedia content is, potentially, a powerful way to improve health literacy | -Geometric mean  -Altmetric score |
| E health tutorial  [40] | **Primary outcomes**  **-Knowledge (**Computer/Internet):  computer/Internet knowledge was significantly higher at post-test than that at pre-test.  **-Skills** in evaluating the quality of online health information websites  The post-test evaluation skills score was significantly higher than the pre-test evaluation skills score  **-** **Procedural skills** in computer/Internet use: Post-test procedural skills score was significantly higher than the pre-test score.  **-eHealth literacy efficacy**  Post-test eHealth literacy efficacy was significantly higher than pre-test eHealth literacy efficacy  **Secondary outcome**  **Attitudes (**Satisfaction with the intervention)  attitudes toward training at post-test approached a statistically significant difference between the multimedia and paper-based conditions, with the OnTOP condition showing more positive attitudes. | -20 items; scoring range 0–20; the higher score, the higher the knowledge.  - 8 items; scoring range 0–8; the higher the score, the better the skills in evaluating the quality of health websites  -12 items; scoring range 0–12; the higher the score, the higher the procedural skill.  **-** The eHealth literacy scale: 8 items, each on a 1–5 Likert scale; scoring range 8–40; the higher the score, the higher the eHealth literacy efficacy  -6 items each on a 1–5 Likert scale; scoring range 6–30; the higher the score, the more positive the attitude |
| Boosting consensus reasoning  [44] | **Primary outcome**  Belief Accuracy: Mean scores of belief accuracy were very high for all waves, a small but significant increase in belief accuracy over time.  -The boosting intervention yielded no significant increase in belief accuracy over the control condition, demonstrating that the boosting infographic was not successful in helping people figure out what is true and what is false.  **Secondary outcome**  Coronavirus-Related Behaviour:  Accurate beliefs were correlated with self-reported behaviour aimed at preventing the coronavirus from spreading. | -Belief Accuracy score: A belief accuracy score was calculated by converting the response to each statement to a number reflecting how accurate the response was; a correct judgment was counted as 1 and an incorrect judgment was counted as –1. A less certain but correct probably true or probably false counted as 0.5 and an incorrect one as –0.5., a don’t know response was counted as 0.  - Agreement with three statements related to Coronavirus-Related Behaviour was measured on a scale from 1 (strongly disagree) to 7 (strongly agree). |
| Accuracy nudging intervention  [43] | **Primary outcome**  **Sharing intension**: sharing intentions for true headlines were significantly higher than for false headlines in the treatment group.  **Secondary outcome:**  The accuracy of the information about COVID-19 on social media: the accuracy nudge makes participants more likely to consider accuracy when deciding whether to share. | -A question about sharing intension answered on a 6-point scale from 1 (extremely unlikely) to 6 (extremely likely).  Sharing intentions were rescaled such that 1 on the 6-point Likert scale was 0, and 6 on the 6-point Likert scale was 1.  -A question about accuracy rating of the headlines (Accurate, not accurate). |
| Educational video [42] | **Primary outcomes**  **Self-care agency**  self-care agency increases over time (not significantly), and at 1 week after the intervention, the video only group is again higher, as the nonsignificant interaction indicated. SCI score remained stable over the 1-week period.  The mean scores for self-care agency were higher in the video-only educational group compared with the video and individual time with HIV nurse clinician group at baseline and 1 week after the intervention. | -Self-as-Carer Inventory (SCI). Self-care agency was measured with the SCI, which is a 40-item, paper-and-pencil self-report of perceived care for oneself. Each item on the SCI is answered on a six-point equal interval scale. The anchors of 1 for very accurate and 6 for very inaccurate. |
| Prior topic knowledge pre-activation support tool  [41] | **Primary outcomes**  **1. Search performance**  Prior knowledge pre-activation was not significant. The effect of age was significant, older adults found fewer correct answers than young adults ones.  2. **Search behaviour**  -**Time spent processing the search engine result pages**: Effects of semantic pre-activation was significant. The effect of age was significant, older adults spent longer time on the search engine result pages.  - **Percentage of time exploring/exploiting navigational paths**: Effects of semantic pre-activation was significant, participants with the semantic pre-activation explored less than participants without the semantic pre-activation. The effect of age was not significant.  -**Percentage of semantically specific keywords used in queries**: Effects of semantic pre-activation was significant, participants with the semantic pre-activation produced queries that contained a larger percent of semantically domain specific keywords (as compared to participants who did not do the semantic pre-activation task. the effect of age on the percentage of semantically specific keywords was not significant. | -To assess search performance, the number of correct answers found was calculated for each search problem.  **Search behaviour measures:**  -The time spent on the search engine result pages corresponded to the time spent analysing the search engine results retrieved by the search engine Google on all the search engine pages  **-** The exploration/exploitation ratio. The number of links opened from Google corresponded to exploration, whereas the number of web pages visited once on that website corresponded to exploitation.  **-** The semantic nature of queries corresponded to the number of keywords produced by participants that were semantically domain-specific (i.e. narrow) or semantically less domain-semantic (i.e. broad). |
