# Supplementary material for: An integrative systematic review on interventions to improve layperson’s ability to identify trustworthy digital health information
Source: PLOS Digit Health. 2024 Oct 25;3(10):e0000638. doi: 10.1371/journal.pdig.0000638 (PMC11508166; doi:10.1371/journal.pdig.0000638)
Supplement: S9 Table — (DOCX) [file pdig.0000638.s011.docx]

**S9 Table: Participant’s characteristics and barriers and facilitators for intervention use by consumers.**

| **Intervention type** | **Study design** | **Sample characteristics** | **Recruitment process/Incentives** | **Barriers for intervention use** | **Facilitators for intervention use** |
| --- | --- | --- | --- | --- | --- |
| Short source evaluation intervention  [37] | Randomised control cross over trial | -48 adults without university education (54.2% female) had various commercial, technical, or handicraft occupations (72.9%), or were housewives, unemployed, or pensioners (27.1%). 25.0% had a lower secondary school degree, 39.6% an intermediate secondary school degree, and another 35.4% an upper secondary school degree. The average age was 43.92 years.  -General Public | Newspaper advertisement/ postings on noticeboards in public places in a mid-size town in southwestern Germany/ Participants were given incentives (Payment) | -Only adult individuals (25 years) without a university or technical college education were allowed to participate.  -At least basic computer and Internet skills were required for participation.  -Medical laypersons were excluded from the study. | -A web-based self-paced training intervention that doesn’t require any additional instructions  - It could be easily made accessible to a broad public. |
| Pharmacy Community Outreach  Program  [45] | Quantitative (questionnaire) | -70 % older adults (Seniors).  -Patients and public | No information/A certificate was awarded to participants upon completion of the learning series. | - Program was held at senior centres settings this reduce the participation from younger adults. | -Pharmacists are well-positioned to engage directly with older adults to address the issues related to older adults’ multiple medications, side effects, and costs given their clinical skills and exposure to high medication costs  -Program was fully funded. |
| Community educational initiatives  [38] | Quantitative (questionnaire) | N=89. Most participants are females 81.8%, more than two-thirds of workshop participants were 45 years or older, residing in Australia 64.6, Tertiary educated 83.5 %, middle and low income 92.1% of good and very good health staus73.4%.  -general public | Study participants were recruited from adults attending any one of the 11 widely advertised free community workshops during March to August 2010/ No incentives | -Unmet demand of workshop materials for education among several tertiary students and health professionals for use of online health information in their research or work practice | -Free interactive workshops.  -Downtown public library setting.  -Small groups of up to 15 people  - All participants found the workshop relevant  - Partnerships among various stakeholders in health |
| Web portal  [2] | Pragmatic randomised controlled parallel trial | -N=96 parents-  -Parents with children under the age of 4-years of age were therefore targeted. At this life stage, parents are typically having many questions about treating and preventing health problems. They are also healthcare users with the highest number of health visits per year for themselves (a mean number of visits per year of 4. 6), and for their children (mean number of visits of 3) [40]. Such parents are also statistically more likely to search for health information online.  - Females 80%, High school 16%, 3+ years of college/ University education 53%.  -Patients and public | Information was distributed at maternity and child health centres, in online advertisements on social media networks, on Internet sites such as Google, and discussion forums for parents. Those who were interested and wished to participate were directed to a recruitment web page. /No incentives | Participants were excluded if anyone else in their household was already participating in the study (to ensure that participants were blinded and to protect against potential sample contamination) and if they did not have children aged 4- years.  Considering that the content of the web portal and tasks included in the trial may be considered as challenging or difficult by many parents, particularly by those with lower health literacy, a higher drop-out could have been expected among those with lower education.  The difference in drop-out between groups may indicate that people joined the study primarily in order to receive access to the portal. Once they had access, they may have dropped out before the first task was given.  the three-day interval between being given access and having to perform the first task, may have been too short to allow them to become familiarised with the portal itself | -interactive online intervention in real life setting  -The web portal was designed from a public health perspective to target both healthy people as well as patients, and to be used either independently or in consultation with health providers.  -It’s content and key intervention targets were informed by extensive literature searches as well as explorative pre-studies with input from people within our target audiences, including focus groups and a questionnaire study based on the Theory of Planned Behaviour (TPB).  -Generic and non-disease specific in focus, the web portal was designed to be applicable to a range of healthcare decisions and settings and included three facilitators or tool-sets to address the main barriers to obtaining information.  -Swine flu was chosen as the subject for the evaluation task, this topic is having considerable potential concern interest to participants. |
| Behavioural intervention  [10] | Randomized clinical trial. | - 835 screened, 527 scheduled for baseline, N=448 baselined.  -The study participants were 320 men, 116 women, and 12 transgender individuals who had used the Internet for any purpose fewer than three times in the month before screening. -The mean age of participants was 42.5 years, and the average number of years of education was 12.2. All participants were fluent in English. Most participants were African American (89%), pants had tested HIV positive. Most participants (80%) reported an annual income below $10,000. Sixty-eight percent of the sample was taking antiretroviral medications | Men and women were recruited from AIDS service organizations, health care providers, social service agencies, and infectious disease clinics throughout Atlanta. Participants received $35 for completing the baseline assessment, with $5 added to the amount of each of the 3-, 6-, and 9-month follow-ups | -Persons who scored below 75% correct on the Test for Functional Health Literacy for Adults (n=18) and Individuals who indicated having used the Internet three or more times in the past month (n =290) were offered enrolment in alternative studies.  - Low reading literacy. | -All participants had to be assured equal, secure, and private Internet access during the study to allow for a viable test of the intervention outcomes. We therefore established a CTAC at an AIDS service agency that also served as the site for the intervention trial.  -The CTAC was located adjacent to the AIDS service agency’s treatment resource library.  -Small group sessions (6-10 participants) with one male and one female community-based group facilitator, at least one of whom was an HIV-positive peer counsellor.  -Adherence to intervention protocols and contamination were monitored in post session briefings and weekly facilitator supervision.  -Context of interactive group discussions, |
| Public library workshop  [9] | Quantitative (questionnaire) | -44 community-dwelling older adults aged 50 to 75. Most participants were born in Canada (17 men; 14 women). Over 90% considered English to be their primary language (n = 41/44). The remaining three individuals also spoke and read German. Participants’ ages ranged from 50 to 75 (mean age: 60.9). One participant withheld her age. All participants had formal education. High school was the highest level of schooling completed by four participants (9.1%). The remaining participants included seven (15.9%) with a community college degree, 9 (20.5%) with some university education, 14 (31.8%) with four years of university education, and 10 (22.7%) with a graduate degree. Most participants were married (n = 32, or 72.7%) and retired (n = 35, or 79.5%). | Recruitment took place in Kitchener-Waterloo-Cambridge and surrounding communities. Recruitment of participants was organized through the Kitchener Public Library (KPL) and the Cancer Prevention and Early Detection Network of Waterloo region (CPEDN-WR). The study was advertised on message boards, print publications, and Web sites. Recruitment was also conducted using other avenues. Flyers and posters were displayed in “seniors” apartment buildings, and recruitment advertisements were placed in community newspapers/No incentives. | -A lot of information in the workshops at one time.  -These workshops were conducted in English with an English-speaking population, lacking cultural diversity. | -The study location, the Kitchener-Waterloo region of Ontario, is a centre for innovative Internet and new information technology initiatives older adults have a positive view of the public library and believe that libraries play an important role as providers of health information.  -A research collaboration was developed between the public library and the local university.  -Computer experience was not required for participation.  -The number of participants per workshop was limited to 15 people to enable a personalized and relaxed training approach. All participants worked at their own computer station that contained the workshop presentation notes and Internet access.  -Using multiple Internet search exercises, these interactive sessions allowed participants to practice search strategies for cancer resources independently and with the assistance of the presenters. |
| Enriching Wikipedia contents [39] | Pragmatic Randomised trial. | N=70 Wikipedia pages, which were highly relevant to up-to-date Cochrane Schizophrenia systematic reviews that contained a Summary of Findings table. | Recruitment was online for Wikipedia pages which are clearly relevant to an up-to date Cochrane Schizophrenia systematic review and that review contained at least one Summary of Findings (SoF) table and considered clear landing page/No incentives | -To some readers, the tabular format was unacceptable as they felt that tables made the pages ‘too academic’ in appearance.  -Six of the 35 intervention group tables were removed after 12 months but the information in the tables remained within the text as did the hyperlinks.  -In this trial, for an outcome to occur, the Wikipedia user had usually to scroll down to find the table, click to expand the drop-down format of the table, seek the reference to that table and finally click out on one of the hyperlinks. This complex set of actions would, we suggest, indicate high levels of motivation to seek further information and it would seem likely that many users of the Wikipedia pages would have not gone further than the initial page. | -The reviews tables were generated using SEED, an open access software, especially created for this study, which uses the original Cochrane review file and rewrites the Cochrane SoF tables in plain English and generates hyperlink references.  -In the design process of the tables, authors communicated with members of ‘Sense about Science’ and consulted publications of the Cochrane Effective Practice and Organisation of Care group in order to increase clarity and readability of the evidence in the tables.  -All content posted in the scope of this trial was sourced from peer-reviewed, systematic reviews published in the Cochrane Library. During the study, 14 of the intervention group’s references had additional hyperlinked PubMed IDs added. The addition of the PubMed IDs broadens the options for gaining additional information for users of the Wikipedia pages.  - Discussions were made with Wikipedia representatives before editing content, as well as using solely verifiable, accessible, and reliable sources. |
| E health tutorial  [40] | Randomized controlled design | 99 community-dwelling older adults (63– 90 years old; mean = 73.09). Of these participants, 91 completed all sessions and the pre- and post-tests (multimedia condition: 45; paper-based condition: 46), resulting in a retention rate of 92%. Female (64.6%), 74 non-Hispanic, English as primary language (91.9%).77% fair-good health status. | Participants were recruited from senior centres, public libraries, and senior-living facilities in Central Texas/ No incentives | - Older adults recruited from geographically limited area  -2 hours sessions are too long due to potential computer-related fatigue.  -OnTOP tutorial was delivered using the conventional computer and keyboard system. | -The tutorial provides instructions presented on top of real, live websites, uses multimedia, is interactive, and enables real time feedback; and derives specific content and features through participatory design, a user-centred approach.  -A trained facilitator (a graduate research assistant) directed participants to use the respective tutorial and provided guidance as needed.  -Each session included no more than eight participants to ensure a small group context.  -Ceiling effect related to the paper-based tutorial, as well as the fact that most of the participants had a high level of education and used the computer/Internet every day with an average computer and Internet use of more than 10 years. |
| Boosting consensus reasoning  [44] | Randomised control trial | -A total of 1212 individuals participated in the study at baseline (T0). A total of 1089 individuals participated in the first follow-up wave (T1), 1070 individuals participated in the second follow-up wave (T2), and 1028 individuals participated in the final wave (T3).  -Female 604 (50.2), 25-34 243 (20.2) ,35-44 209 (17.4), 45-54 199 (16.6), 55-64 232 (19.3), 65-74 139 (11.6) ,≥75 16 (1.3)  White 918 (76.4) | Prolific was used for recruitment, a UK-based online crowdsourcing platform/ Incentives were given at baseline and follow up waves. | -As preregistered, participants who failed the attention check at T0 were excluded and replaced.  -Participants who indicated at the T0 seriousness check that their data should not be used were excluded from further participation and their data were not used, but they were not replaced. | -The infographic set out three steps that can be used to evaluate a claim: (1) searching for a statement indicating consensus among scientists, (2) checking the source of this consensus statement, and (3) evaluating the expertise of the consensus. |
| Accuracy nudging intervention  [43] | Randomised control trial | In total, 1,145 participants began the study. However, 177 did not indicate using Facebook or Twitter and therefore did not complete the survey. A further 112 participants did not complete the study. The final sample consisted of 856 participants (mean age = 47 years, age range = 18–86; 385 men, 463 women, and 8 who responded “other/prefer not to answer”). | Participants were recruited using Lucid, an online recruiting source that aggregates survey respondents from many respondent providers/ No incentives | Participants indicating not using Facebook or Twitter. | -The intervention framework was designed as pre-test. An advantage of this design is that the manipulation is subtle and not explicitly linked to the main task. Thus, it is unlikely that any between-conditions difference was driven by participants’ believing that the accuracy question at the beginning of the treatment condition was designed to make them take accuracy into account when making sharing decisions during the main experiment. |
| Educational video [42] | Quasi-experimental | -Low-income persons living with HIV/AIDS (N = 100).  -Age 49.95 (±9.72), Male 59 (59%) Race African American 82 (82%) Non-Hispanic/ Latino 84 (84%), More than HS 58 (58%). | Recruitment occurs at two locations of an adult treatment program located in New York/No incentives. | -The time between the intervention and the measurement of self-care agency might have been too short and the intervention too brief.  -Video was conducted in English | -Participants were accepted for the study if they were members of the HIV treatment program, were 18 years of age or older, were HIV-positive, were able to provide informed consent to be a research participant and spoke English.  -Participants reported enjoying completing the SCI questionnaire, saying that it gave them an opportunity to look at themselves and their health.  -An HIV expert nurse clinician conducted the educational interventions |
| Prior topic knowledge pre-activation support tool  [41] | Quantitative (survey) | 48 participants. twenty-six young (18 to 32 years) and twenty-two older adults (60 to 77 years), predominantly females, English speakers. Older adults were all retired, in good health. Both groups had completed upper college studies. | Recruited via the news e-magazine of the University of Illinois at Urbana-Champaign (USA)/Incentives given to participants. | -The health questions tackled knowledge about how the human body functioning and not health issues (for which older adults might have more knowledge about).  - The search problems designed were a priori complex, in order to foster reformulation and web navigation. the search problems required users to engage cognitive resources to make inferences, assess information relevance and select websites to visit. | - |
