# Supplementary material for: An integrative systematic review on interventions to improve layperson’s ability to identify trustworthy digital health information
Source: PLOS Digit Health. 2024 Oct 25;3(10):e0000638. doi: 10.1371/journal.pdig.0000638 (PMC11508166; doi:10.1371/journal.pdig.0000638)
Supplement: Attachment — Submitted filename: Response to reviewers systematic review.docx [file pdig.0000638.s012.docx]

**Response to reviewers**

Please find point by point response to reviewer, all the changes are highlighted in grey in the main paper.

**1.      The title is clear but somewhat generic. Consider specifying the target audience or health context to make it more specific.**

The title has been updated, and the target audience are specified as follows:

“An integrative systematic review on interventions to improve layperson’s ability to identify trustworthy digital health information.

**2.      The introduction Identifies the significance of health literacy and digital health literacy in accessing reliable information. However, there are a few aspects that could be improved:
•       The introduction lacks a specific and focused research gap or problem statement.**

The introduction has been updated to be more specific and covers a problem statement and research gap

**“Problem statement**

Barriers to consumer use of online health information include lack of physical access to the computer and internet, motivation, and a real or perceived lack of knowledge, cognitive skills to find and use information (12–15). Of these, motivation and skills are critical. Simply increasing access to online information will not lead to increased use unless consumers are motivated and able to make effective use of the internet and the information they find (16). The expectation that online health information will be useful is one of the strongest predictors of internet use, and a lack of skills is debated as one possible reason for not using the internet. Specifically, potential users may lack the skills to effectively locate, evaluate and use online health information depending on several factors (17–19).

Previous studies identified many critical factors in determining consumer's digital information-seeking behaviour, such as demographic characteristics (age, gender, educational status, socioeconomic class, and employment status) and health status (20). Older adults are reluctant to use the internet, which is consistent with offline gender-based health-seeking behaviour (21). Individuals who are better educated, employed, and of a high socioeconomic class prefer to use websites as a source of health information (22). Those with poor/ fair health, chronic conditions, and long-term disability are more likely to visit health websites than those who reported better health (23). A study targeting rural digital immigrants in China (24) reported that lack of health information literacy and low readability of digital health information are significant barriers for digital immigrants seeking and evaluating online health information.

Evaluating the trustworthiness of digital health information has been a critical issue in consumer health informatics research for many years (25). A recent review done in Finland (2020) showed that participants face many challenges in evaluating the reliability of online health information (26). Although they understand that online information should not always be trusted, they remain unsure of how to evaluate its credibility (27–29). Another study by Metzgar et al. (2007) reported that internet consumers rely on either central or peripheral strategies for health information evaluation depending on high or low motivational status, respectively (30).”

**Research gap**

“To our knowledge only two reviews have been conducted since 2010 to explore interventions targeting lay people. Car et al (2011) conducted a systematic review on interventions for enhancing consumers’ online health literacy and found two interventions (16). This was followed by Lee et al. comprehensive review (2014) on interventions to assist health consumers to find reliable online information which reported seven publications (31). This review was unable to follow systematic review methods due to the paucity of research and humanistic interventions reported. “

**•       Limited clarity on the primary objective or hypothesis of the study.**

The aim and objectives of the study are amended as follows:

**Aim**

To explore the useability and impact of interventions designed to improve laypersons' ability to identify trustworthy health-related online information.

**Objectives**

1. Outline the interventions which are designed to improve laypersons' ability to identify trustworthy online health-related information.

2. Identify the barriers and facilitators for using the interventions by laypersons.

3. Understand the interventions effects on layperson's knowledge, skills, attitudes, and confidence to identify trustworthy online health-related information.

4. Understand the interventions effects on layperson’s health outcomes.

The hypothesis of the study is added to the introduction as follows:

“Based on the Information, Motivation and Behavioural Skills (IMB) model, we hypothesise that enhancing consumers' online health literacy has a positive effect on health behavioural outcomes.

**•       The background section is broad and lacks specificity.**

The background section has been updated to be more specific. It includes problem statement, research gap, types of implemented interventions, what the previous reviews explored, why our review is different, and our hypothesis.

“The active involvement of healthcare consumers in the co-management of their own health is an essential component of sustainable health and healthcare and central to evidence-based practice (1). However, effective participation is dependent on access to reliable information, and acquiring the knowledge, motivation, and competence to assess, understand, evaluate, and act upon the information available (2). The World Health Organization described health literacy as the main desired outcome of health education, as an asset in itself, and as a public health issue (3).

There is growing evidence that health consumers increasingly rely on the internet for health information (6,7). The internet offers a powerful means for obtaining information about medical conditions and their treatments (7). A recent study by Morley et al. in 2020 has recognised the infosphere as a social determinant of health (3). It was included in Dahlgren and Whitehead's model of health determinants (8). Using this model, the determinants are the general socioeconomic, cultural, environmental, and now informational conditions (3).

Digital health literacy is the ability to use information and communication technologies to find, evaluate, create, and communicate health information, requiring both cognitive and technical skills (4). Digital health literacy skills include computer and internet literacy, information literacy, functional literacy, and navigational and critical skills literacy (5). Computer literacy refers to the ability to operate in computer-supported environments and includes both technical competence and an understanding of how information is presented electronically. Information literacy is “knowing when and why you need information, where to find it, and how to evaluate, use and communicate it”.

Barriers to consumer use of online health information include lack of physical access to the computer and internet, motivation, and a real or perceived lack of knowledge, cognitive skills to find and use information. Of these, motivation and skills are critical. Simply increasing access to online information will not lead to increased use unless consumers are motivated and able to make effective use of the internet and the information they find. The expectation that online health information will be useful is one of the strongest predictors of internet use, and a lack of skills is debated as one possible reason for not using the internet. Specifically, potential users may lack the skills to effectively locate, evaluate and use online health information.

Although the Internet offers many advantages to online health information consumers, searching and evaluating specific information can cause many challenges for the lay public (17–19). Metzgar et al. (2007) reported that internet consumers rely on either central or peripheral strategies for health information search and evaluation depending on high or low motivational status, respectively (30). For instance, internet users typically use suboptimal searching and evaluation strategies (16). A recent review done in Finland (2020) showed that participants face many challenges in evaluating the reliability of online health information (26). Although they understand that online information should not always be trusted, they remain unsure of how to evaluate its credibility (27–29).

As such, many interventions are implemented targeting layperson's ability to identify reliable online information such as educational programs, interactive workshops, health literacy curricula, community outreach programs, and online portals. All those interventions aim to teach people how to navigate online and then measure the change in knowledge and internet skills for the purpose of evaluation.

To our knowledge only two reviews have been conducted since 2010 to explore interventions targeting lay people. Car et al (2011) conducted a systematic review on interventions for enhancing consumers’ online health literacy and found two interventions. This was followed by Lee et al. comprehensive review (2014) on interventions to assist health consumers to find reliable online information which reported seven publications (31). This review was unable to follow systematic review methods due to the paucity of research and humanistic interventions reported.

We aim to carry out a new systematic review of the topic given that the internet has changed a lot since the last review was carried out. To our knowledge, our systematic review paper is the first to explore the implemented interventions for lay people and their effects on e-health literacy skills and beliefs, and health outcomes.

Based on the Information, Motivation and Behavioural Skills (IMB) model, we hypothesise that enhancing consumers' online health literacy has a positive effect on health behavioural outcomes. This hypothesis is in line with research demonstrating that both participation in educational training and use of the internet for health information are associated with positive health outcomes.”

**•       The research questions could be more precise and directly tied to the objectives.**
The research questions have been amended and directly tied to the objectives as follows:

1. What are the types of interventions to improve laypersons' ability to identify trustworthy digital health information?
2. What are the barriers and facilitators for using the interventions by laypeople?
3. What are the interventions effects on layperson's Knowledge, skills, attitudes, and beliefs to identify trustworthy online health-related information?
4. What are the interventions effects on layperson’s health outcomes? **3.      The methods section demonstrates a systematic and comprehensive approach to study selection, data extraction, quality assessment, and data synthesis. However, potential challenges arise from the inclusion of diverse study designs, multiple languages, and the broad study setting, which may affect the synthesis and generalizability of findings. Transparent reporting and addressing potential biases in the synthesis process are recommended.**
   Details on how the quality assessment tool was used has been added to the quality assessment section. Transparent reporting and addressing the risk of bias in the synthesis process has been added to the following sections.

**“Quality Assessment”**

“The mixed methods appraisal tool (MMAT) (version 18) was adopted to integrate quantitative, qualitative, and mixed-methods evidence. This tool is comprised of two screening items, followed by 15 appraisal items in three sections, including (1) five items on the Randomised control trials, (2) five items on the quantitative descriptive component, (3) five items on the non-randomised studies. Reviewers independently evaluated the risk of bias of all selected articles with the MMAT, on an item-by-item basis. There are three response options: 'Yes' meaning the criterion is met, 'No' meaning the criterion is not met, and 'Can't tell' when there is not enough information in the paper to judge whether the criterion is met. Responding ‘No’ or ‘Can’t tell’ to one or both screening items indicates that the paper is not an empirical study, and thus couldn’t be appraised using the MMAT. Since there are only a few items for each domain, the score was presented using descriptors (%): 100% quality criteria met, 80% quality criteria met, 60% quality criteria met, 40% quality criteria met, 20% quality criteria met. The quality of the paper is rated good, moderate, or poor when 100% of quality criteria are met, 80% or 60% of quality criteria are met, 40% or 20% of quality criteria are met, respectively (35).”

“The MMAT was used to assess the quality of studies with different methodological designs. Two reviewers independently appraised the quality of each included study. Disagreements between reviewers were resolved by discussion. Rating ‘Can’t tell’ to the appraisal items has led the reviewers to look for companion papers and contact the authors for more information and clarification. If the information is complete to address the item, the reviewers agreed to rate the item as “Yes”; otherwise, we assigned a “no” response. However, none of the review authors is methodologically expert in all study designs, which may have introduced a degree of bias”.

**“Data Extraction”**

“Particular results of the intervention outcomes were not reported at all (‘selective non-reporting of results’) or were reported incompletely (‘selective under-reporting of results, e.g., stating only that “P>0.05” rather than providing summary statistics or an effect estimate and measure of precision), introducing a selective (non-) reporting bias. Some authors have been contacted to provide complete results; no replies have been received.”

“**Strength and limitations”**

“Per the protocol, we plan to search comprehensively and include studies in different languages. However, upon selection, neither Spanish nor Portuguese studies were found. As such, we included only English studies; this could lead to language bias. The included studies reported interventions implemented in different country settings; this may introduce a location bias and affect the generalisability of our findings.”

**4.      The Results section provides an overview of the study's process, including the identification of records, screening, and inclusion/exclusion criteria. However, there are areas that could be improved:
•       The section on quality assessment could be clearer. Consider providing a concise summary of the key findings, focusing on methodological strengths and weaknesses of the included studies.**

The section on the quality assessment has been amended to be clearer and provide a concise summary of the key findings.

“A total of two authors independently appraised the twelve articles that met the inclusion criteria for methodological quality. The S7 table summarises the questions answered in the mixed method appraisal tool checklist (version 18) (46). Of the twelve included studies, only two studies (16.7%) scored well on the quality assessment (100% of the quality criteria met), including one quantitative study (38) and a quasi-experimental (non-randomised control trial) design (42). The rest of the studies 10/12 (83.3%) scored moderate, with 60% and 80% of the quality criteria met, including all the randomised control trials (7/12) (2,10,37,39,40,43,44) and the remaining three quantitative study designs (9,41,45) . All the randomised control trials were rated of moderate quality as they didn’t report if the outcome assessor is blinded to the intervention provided (2,10,37,39,40,43,44) , and half of them didn’t mention if the randomisation is appropriately performed (37,34,44) , which could introduce performance and selection bias, respectively, and affect the synthesis and generalisability of our findings. We contacted authors for the missing information, but no replies were received.”

- **The discussion of biases could be expanded to provide more insights into the potential impact on the study's findings and the overall quality of evidence.**

The above-mentioned point has been addressed in the discussion as follows:

“All the randomised control trials were rated of moderate quality as they didn’t report if the outcome assessor is blinded to the intervention provided (2,10,37,39,40,43,44) , and half of them didn’t mention if the randomisation is appropriately performed (37,34,44) , which could introduce performance and selection bias, respectively, and affect the synthesis and generalisability of our findings. We contacted authors for the missing information, but no replies were received.”

**•       Consider concluding the Results section with a brief summary of the main findings, which could serve as a transition to the subsequent sections of the paper.**

A brief summary of the main findings to serve as a transition to the subsequent sections of the paper has been added.

“Media literacy interventions are the most common type of the implemented interventions. Few studies measure the effect of the intervention on health outcomes, the minority is significantly effective. All the procedural and navigation/ evaluation skills building interventions are effective with statistically significant results. The main barriers for layperson’s participation in web-based interventions are lack of participant’s computer/internet experience and absence of guidance/facilitator. Few interventions are delivered in a suitable location for the participant’s, in addition the minority involved lay people in designing the intervention.”

**5.      The discussion outlines the study's findings and attempts to draw meaningful conclusions. However, there are several aspects that could be criticized and improved:
•       The use of subheadings within the discussion section could enhance the structure and make it easier for readers to navigate through different aspects of the study.**

Subheadings have been added to the discussion as follows:

**1. Summary of main results**

**2. Agreements and disagreements with other studies**

**a. Intervention types**

**b. Interventions effects on health outcomes**

**c. Intervention style effectiveness**

**d. Barriers to intervention use**

**e. Facilitators of intervention use**

**3. Potential biases**
**•       Some points are repeated, such as the emphasis on the dominance of media literacy interventions. It's essential to avoid unnecessary repetition and focus on presenting new insights or reinforcing critical points more effectively.**

The above-mentioned point has been addressed in the relevant paragraphs. The text was reorganised to avoid repetition and improve organisation and flow.

“The dominance of media literacy intervention was reported in studies done by Car et al. and Lee et al. in UK and Australia (2011, 2014), respectively (16,31). Both publications explored exclusively media literacy interventions. However, the trend of interventions has changed during the last five years (2018-2023) focusing on competence-based, top-down, and problem-based search learning approaches. Our paper is the first to explore those types. However, more evidence is needed to determine the effectiveness and scalability of such interventions.”

**•       The discussion could benefit from a more critical evaluation of the quality and limitations of the studies reviewed. Discuss the methodologies used in the studies, potential biases, and the generalizability of the findings.**

Please refer to the text below addressing the above-mentioned point.

**“Potential biases**

The MMAT is used to assess studies with different methodological designs. None of the quality assessment reviewers are experts in all types of study designs. Most of the included studies have a randomised control trial study design; all were rated of moderate quality as they didn't report blinding of the outcome assessor to the intervention and if the randomisation is appropriately performed. This could introduce performance and selection bias, affecting the synthesis and generalisability of our findings. Thus, there is a need for well-designed and rigorously conducted randomised controlled trials to understand the effectiveness of the interventions for improving digital health literacy. As our review includes complex interventions, outcomes are likely affected by participant's characteristics such as age, health status, and aspects of the intervention (for example, mode of delivery or setting).”

**•       There are instances where the findings are presented as contradictory, such as the disagreement with Car et al. (2011). Clarify the reasons for these inconsistencies and discuss potential factors contributing to differing results.**

The relevant paragraph has been updated and reasons has been added as follows:

**“Interventions effects on health outcomes**

We also noted few studies measuring the impact of digital health literacy interventions on the patients’ health outcomes (10, 42, 44, 45); the minority are effective (10). This is supported by a study done recently in Australia (2021) on the effects of web-based portals on parental health knowledge and child health outcomes (47). This disagrees with Car et al.'s (2011) study findings (16), as the two included studies measured the participants' health outcomes, and half of them were statistically effective.”

The potential factors contributing to differing results are discussed below:

“Our review reported the implemented interventions since 2006 and conversely explored twelve studies, third have measured the health outcomes, however, only one intervention is statistically effective. In addition, it is hard to compare across the studies due to differing baseline health literacy in different context.”

**6.      Conclusion:
•       Could be more succinct and highlight the key takeaways from the study. Summarize the main findings and their implications for future research or practical applications.**

The main findings and their implications for future research has been summarised in the conclusion.

**“Conclusion**

Few interventions reported using competence building approaches to teach people how to navigate online information. Most of the included studies focused on measuring digital health literacy skills and attitudes rather than the long-term effect of the intervention on participant’s health outcomes. Only a handful of studies demonstrated improved health. One important finding of our study is that limited computer/internet skills are still a major barrier to using web-based interventions. We found a lack of descriptions of if and how end users were involved in intervention design. Few studies are noted to use design thinking approaches to facilitate intervention use by consumers. Future work could tailor interventions and context to participants needs. Designing future educational strategies with involvement of end users could play an essential facilitating role. There is considerable scope for further research to understand who is best involved in the design process. Implementing a practical educational intervention in a location convenient to the participants could facilitate intervention use by consumers. However, more evidence is needed to understand where the best place is to do these classes and when is the best time. Program facilitators could play a critical role in guiding laypeople how to navigate online health information. However, more research is needed to explore who is best placed to do this. Moreover, equity driven interventions could help to address socioeconomic disadvantaged (elderly) needs considering implementation of digital health literacy interventions.”

**•       While the implications for practice are mentioned, they could be more explicit and actionable. Provide concrete recommendations for practitioners or policymakers based on the study's findings.**

Concrete recommendations for practitioners or policymakers based on the study's findings has been provided.

**“Implications for practice**

Educational strategies to support people in improving their digital health literacy, focusing on addressing computer and internet illiteracy, involving facilitators for intervention delivery, and focusing on competence building and design thinking approaches, could have promising outcomes. Providing access to the computer/ internet without training is not sufficient to improve digital health literacy. This has implications for wider policy and suggests that investment in infrastructure needs to be accompanied by investment in training. Many potential agents must join forces to build the health literacy of the lay public- healthcare professionals, health service organizations, community centres, public libraries, and continuing education providers.”

**•       Enhance the conclusion by proposing specific directions for future research based on the identified gaps and limitations.**

Specific directions for future research based on the identified gaps and limitations have been provided in the conclusion.

“Future work could tailor interventions and context to participants needs. Designing future educational strategies with involvement of end users could play an essential facilitating role. There is considerable scope for further research to understand who is best involved in the design process. Implementing a practical educational intervention in a location convenient to the participants could facilitate intervention use by consumers. However, more evidence is needed to understand where the best place is to do these classes and when is the best time. Program facilitators could play a critical role in guiding laypeople how to navigate online health information. However, more research is needed to explore who is best placed to do this. Moreover, equity driven interventions could help to address socioeconomic disadvantaged (elderly) needs considering implementation of digital health literacy interventions.”
